# Metabolic disorders induced by *PNPLA3* and *TM6SF2* gene variants affect chronic kidney disease in patients infected with non-genotype 3 hepatitis C virus

**DOI:** 10.1186/s12944-023-01858-4

**Published:** 2023-07-03

**Authors:** Jia Liu, Wenqian Qi, Song Wang, Yonggui Zhang, Xu Wang, Derong Sun, Yanhui Xu, Jingyi Shi, Honglei Duan, Qian Zhang, Hongguang Wang, Jiangbin Wang

**Affiliations:** 1 Department of Gastroenterology and Hepatology, China-Japan Union Hospital of Jilin University, No.126 Xiantai Street, Changchun, Jilin Province 130033 China; 2 Department of Urology, the First Hospital of Jilin University, Changchun, Jilin Province 130021 China; 3 Department of Gastroenterology, The Fourth Hospital of Harbin Medical University, Harbin, 150001 China; 4 Department of Gastroenterology, Jilin City People’s Hospital, Jilin, 132001 China

**Keywords:** Chronic HCV infection, PNPLA3, TM6SF2, Chronic kidney disease

## Abstract

**Background:**

Patients with chronic hepatitis C virus (HCV) infections differ in their risk for metabolic disorders and chronic kidney disease (CKD). The aim of this study was to investigate the effect of metabolic disorders induced by genetic factors on CKD in HCV-infected patients.

**Methods:**

Patients with chronic non-genotype 3 HCV infection with or without CKD were examined. *PNPLA3* and *TM6SF2* variants were determined using high-throughput sequencing. The relationships of variants and different combinations with metabolic disorders were analyzed in CKD patients. Univariate and multivariate analyses were used to identify factors associated with CKD.

**Results:**

There were 1022 patients with chronic HCV infection, 226 with CKD and 796 without CKD. The CKD group had more severe metabolic disorders, and also had higher prevalences of liver steatosis, the *PNPLA3* rs738409 non-CC genotype, and the *TM6SF2* rs58542926 CC genotype (all *P* < 0.05). Relative to patients with the *PNPLA3* rs738409 CC genotype, patients with the non-CC genotype had a significantly decreased eGFR and a greater prevalence of advanced CKD (CKD G4-5). Patients with the *TM6SF2* rs58542926 CC genotype had a lower eGFR and a higher prevalence of CKD G4-5 than those with the non-CC genotype. Multivariable analysis indicated that multiple metabolic abnormalities, including liver steatosis and the *PNPLA3* rs738409 C > G variant, increased the risk of CKD, but the *TM6SF2* rs58542926 C > T variant decreased the risk of CKD.

**Conclusion:**

Specific *PNPLA3* rs738409 and *TM6SF2* rs58542926 variants are independent risk factors for CKD in patients with chronic HCV infections and are associated with the severity of renal injury.

## Background

Hepatitis C virus (HCV) infection is a global public health problem that has an estimated overall prevalence of 2.5% [1]. Our previous survey of the physical examinations of nearly 230,000 people in Jilin province from 2010 to 2013 showed that 2.98% of these individuals were positive for anti- HCV antibodies, indicating that chronic HCV infection is a serious public health issue in China. In addition to hepatitis, liver fibrosis, cirrhosis, and even hepatocellular carcinoma, HCV infection can also cause extrahepatic complications [2, 3]. Approximately 40 to 76% of patients with chronic HCV infections have at least one HCV-related extrahepatic manifestation at presentation, such as rheumatoid arthritis, Sjögren’s syndrome, autoimmune thyroiditis, membranoproliferative glomerulonephritis, membranous nephropathy, and idiopathic pulmonary fibrosis [4–6].

Epidemiological studies have found that that chronic HCV infection is associated with chronic kidney disease (CKD) and promotes the progression of CKD to end-stage renal disease [7]. A 2017 meta-analysis showed that individuals with positive serum anti-HCV antibodies had a higher risk of CKD, and that this positivity was an independent predictor of death in dialysis patients [8]. Some studies suggested that interferon or direct-acting antiviral (DAA) treatment can delay the progression of CKD [9, 10]. However, other studies showed that DAA treatment does not reduce the incidence of CKD in patients with HCV infection, and that about 15% of patients with HCV infection complicated with CKD do not show improvement [11, 12]. These results suggest that besides viral factors, there are other risk factors for developing CKD in HCV infected patients. Identification and therapeutic targeting of these additional factors may help to improve the prognosis of patients with chronic HCV infections.

CKD in these patients, which is mainly attributed to cryoglobulinemia and the deposition of HCV antigen-antibody complexes in the glomeruli, is a major extrahepatic complication. However, evidence has shown that anti-HCV treatment does not significantly decrease the risk of cryoglobulinemia [12]. Recent epidemiological studies also suggested that HCV infection can lead to lipid metabolism disorders and insulin resistance, similar to non-alcoholic fatty liver disease (NAFLD), and thereby induce CKD. Moreover, the co-occurrence of HCV infection and NAFLD leads to exacerbation of both diseases [2, 3, 13–16]. However, it remains unclear why patients infected with the same HCV genotype differ in lipid metabolism disorders, and why they have prognostic differences in renal function after successful anti-HCV therapy.

Recent research reported that single nucleotide polymorphisms (SNPs) in two genes, patatin-like phospholipase domain-containing protein 3 (*PNPLA3*) and transmembrane 6 superfamily member 2 (*TM6SF2*), increased the risk of NAFLD [17]. In particular, the rs738409 SNP of *PNPLA3* (I148M) and the rs58542926 SNP of *TM6SF2* (E167K) are independent risk factors for liver steatosis and steatosis severity in patients with HCV infections [18, 19]. PNPLA3 regulates intracellular lipid metabolism through its triglyceride (TG) hydrolase activity, and the rs738409 SNP of this gene leads to decreased lipohydrolase activity and deposition of TGs in the liver [20, 21]. TM6SF2 regulates liver lipid metabolism, and the rs58542926 SNP of this gene leads to misfolding of the protein and a decreased level of the protein due to increased degradation, followed by accumulation of TGs and cholesterol in the liver [22, 23]. Inhibition of TM6SF2 expression in human Huh7 and HepG2 cells reduces the expression of PNPLA3 [24], suggesting that a direct or indirect interaction of these proteins may aggravate intrahepatic lipid deposition and the progression of liver steatosis. There is also evidence that these two SNPs are associated with CKD in patients with NAFLD [25]. However, no studies have yet examined the relationship of these two SNPs with liver steatosis and their contribution to CKD in patients with chronic HCV infections.

In addition to these human genetic variants, the HCV genotype can also affect liver steatosis in patients with HCV infections. In particular, viral factors are considered major risk factors for liver steatosis in patients with infections by the HCV genotype 3, and metabolic factors (such as insulin resistance and lipid metabolism disorders) are considered major risk factors for liver steatosis in patients infected by the other HCV genotypes. Because non-genotype 3 HCV infections are most prevalent in Northeast China, this led to the hypothesis that metabolic disorders induced by genetic factors may be related to HCV-associated kidney injury.

Therefore, the aim of this work was to investigate risk factors for renal injury in patients from Northeast China who have chronic HCV infections. Thus, patients with chronic non-genotype-3 HCV infections were examined and the genotype and allele frequencies of the rs738409 SNP in *PNPLA3* and the rs58542926 SNP in *TM6SF2* were determined. Then, the genotype and allele frequencies in patients with and without CKD were compared, their relationships with indicators of metabolic abnormalities were examined, and the estimated glomerular filtration rate (eGFR) of these CKD patients was determined.

## Methods

### Patients

This retrospective study was conducted in four locally representative liver disease centers in Jilin and Heilongjiang provinces (Northeast China): China-Japan Union Hospital of Jilin University and Jilin City People’s Hospital (central region), People’s Hospital of Hunchun City (eastern region), and the Fourth Affiliated Hospital of Harbin Medical University (northern region). All enrolled patients were HCV treatment-naive and were admitted to one of these hospitals from 2016 to 2019 with chronic HCV infections, anti-HCV positivity, and HCV RNA viral loads of at least 15 IU/mL. Chronic hepatitis C was diagnosed according to EASL Recommendations on Treatment of Hepatitis C [26]. The exclusion criteria were: (*i*) consumption of excessive alcohol (> 30 g/day for men and > 20 g/day for women); (*ii*) positivity for hepatitis B virus (HBV) surface antigen or HBV DNA; (*iii*) positivity for serum anti-human immunodeficiency virus; (*iv*) an autoimmune liver disease, based on auto-antibody tests and liver and bile system imaging; (*v*) use of drugs that can cause liver steatosis (e.g., valproic acid, glucocorticoids, tetracycline, amiodarone, maleic acid), or lipid-lowering drugs, or drugs associated with renal toxicity (e.g., high-dose non-steroidal anti-inflammatory drugs, aminoglycoside antibiotics, cyclosporin A, and Chinese herbal decoctions) during the previous 6 months; (*vi*) complication with polycystic ovary syndrome, Wilson’s disease, hypothyroidism, or another metabolic disease that can cause liver steatosis; (*vii*) decompensated cirrhosis; (*viii*) cancer; or (*ix*) a history of kidney transplantation.

CKD and the categories of CKD were defined according to the 2012 clinical practice guideline of the Kidney Disease: Improving Global Outcomes [27]. In particular, patients diagnosed with CKD had at least one of the following conditions for more than 3 months: (*i*) albuminuria (albumin excretion rate [AER] ≥ 30 mg/24 h; urine albumin-to-creatinine ratio ≥ 3 mg/mmol or ≥ 30 mg/g); (*ii*) urine sediment abnormalities; (*iii*) electrolyte and other abnormalities due to renal tubular disorders; (*iv*) abnormal structural findings based on imaging; or (*v*) eGFR below 60 mL/min/1.73 m^2^, determined using the Chronic Kidney Disease Epidemiology Collaborative (CKD-EPI) equation [28]. Category G1 was defined as an eGFR of at least 90 mL/min/1.73 m^2^; category G2 as an eGFR of 60 to 89 mL/min/1.73 m^2^; category G3 as an eGFR of 30 to 59 mL/min/1.73 m^2^; category G4 as an eGFR of 15 to 29 mL/min/1.73 m^2^; and category G5 as an eGFR below 15 mL/min/1.73 m^2^. All patients provided venous blood samples for biochemical, virological, and genetic analyses and general clinical information and imaging findings were recorded. The study protocol was approved by the Ethics Committee of the individual centers (No: 2016ks021) and all participants provided written informed consent.

### Evaluation and staging of liver steatosis and fibrosis

Liver steatosis was evaluated using color Doppler ultrasound, computed tomography (CT), and transient ultrasound elastography (Fibroscan, Echosens, French). Liver steatosis was graded using the controlled attenuation parameter (CAP) and the criteria of Karlas et al. [29] as grade S1 (CAP 248–267 dB/m), grade S2 (CAP 268–279 dB/m), or grade S3 (CAP ≥ 280 dB/m).

Liver fibrosis was evaluated using transient ultrasound elastography and the results were reported as the liver elastic modulus in kilopascals (kPa). The WFUMB guidelines and recommendations for clinical use of ultrasound elastography [30] were used to classify the results as nil-mild fibrosis (2.5–7.0 kPa), moderate to severe fibrosis (7.1–12.5 kPa), or cirrhosis (> 12.5 kPa).

### Laboratory tests

All four liver centers used the same standards for sample collection and data analysis. For all participants, routine urine and serum biochemical indicators were determined using an automated biochemical analyzer (AU5800, Beckman Coulter Inc., Brea, CA). All venous blood samples were obtained after 12 h of overnight fasting. For determination of insulin resistance, measurements of fasting plasma glucose (FPG) in mmol/L (AU5800) and fasting insulin (FINS) in µIU/mL (Elecsys 2010; Roche Diagnostics, Indianapolis, IN) were entered into the homeostatic model assessment for insulin resistance:

### HOMA-IR = (FPG × FINS)/22.5

#### Genotyping

HCV RNA genotyping was performed by gene sequencing using a Roche Light Cycler 480 (Roche Molecular Diagnostics, Alameda, CA) and an ABI 310 (Applied Biosystems, USA). HCV RNA levels were quantified using the reverse transcription quantitative polymerase chain reaction (RT-qPCR) with the COBAS AmpliPrep/COBAS TaqMan HCV Test, whose detection limit is 15 IU/mL (48 Analyzer, Roche).

PCR and high-throughput sequencing were used to detect *PNPLA3* rs738409 and *TM6SF2* rs58542926 variants with the following primers: 5’-CAGCCAGTTTACCTTACAGATAGC-3’ (forward) and 5’-GTCCGAGGGTGTATGTTAGTTCC-3’ (reverse) for *PNPLA3* rs738409; 5’-GGAAAGTTCAGGCACATTGG-3’ (forward) and 5’-AGCCTGGGTGACAGAGCAAG-3’ (reverse) for *TM6SF2* rs58542926. Two-step PCR was used to amplify the sequence containing each target SNP and to prepare a compatible Illumina sequencing library. Finally, the PCR product was purified and recovered using AMPure XP beads, and the libraries were then quantified and pooled. Paired-end sequencing of the library was performed on HiSeq XTen sequencers (Illumina, San Diego, CA).

### Statistical analysis

All statistical analyses were performed using SPSS version 22.0. The genotype frequencies were tested for Hardy-Weinberg equilibrium (HWE). Qualitative data were compared using Chi-square test or Fisher’s exact test, as appropriate. Quantitative data were presented as medians and interquartile ranges (IQRs) and compared using the rank sum test. Variables with non-normal distributions (i.e., hepatitis C viral load) were log-transformed before analysis. Univariate and multivariate logistic regression analyses were used to identify factors associated with chronic HCV infection complicated with CKD. A *P* value below 0.05 was considered statistically significant.

## Results

### Characteristics of the CKD and non-CKD groups

The records of 1022 patients who had chronic non-genotype 3 HCV infections from 2016 to 2019 were examined (Table 1). Most patients (63.1%, 645) were infected with HCV genotype 1 and 226 (22.1%) of them had CKD. The CKD group had significantly higher body mass index (BMI), HOMA-IR, serum total cholesterol (TC), TGs, low-density lipoprotein cholesterol (LDL-C), C-reactive protein (CRP), liver CAP, and prevalences of liver steatosis and history of hypertension, but a significantly lower eGFR (all *P* < 0.05). The two groups had no significant differences in any other measured variables, especially severity of liver steatosis (all *P* > 0.05). The genotype distribution of SNP rs738409 in each group was consistent with HWE. The genotype distribution of SNP rs58542926 was consistent with HWE in the CKD group, but deviated from HWE in the non-CKD group (data not shown).

**Table 1:** Characteristics of patients in the CKD and non-CKD groups.^*^

Characteristic	CKD (n = 226)	non-CKD (n = 796)	*P* value	
Age, years	44.0 (39.25, 50.00)	44.0 (39.00, 49.00)	0.269	
male/female, %	57.1/42.9	53.0/47.0	0.279	
History of hypertension^**^, n (%)	57 (25.2)	58 (7.3)	**< 0.001**	
eGFR, mL/min/1.73 m^2^	36.96 (25.75,49.58)	94.33 (81.57,104.74)	**< 0.001**	
HCV genotype 1/2, %	62.8/37.2	63.2/36.8	0.921	
HCV load, log IU/mL	5.0 (5.0,6.0)	5.0 (5.0,6.0)	0.109	
Metabolic profile				
BMI, kg/m^2^	25.45 (22.95,27.60)	24.52 (21.59,27.16)	**< 0.001**	
HOMA-IR	2.40 (1.95,3.48)	2.21 (1.77,2.55)	**< 0.001**	
TC, mmol/L	5.76 (4.83,6.39)	5.38 (4.66,6.01)	**0.001**	
TG, mmol/L	2.36 (2.08,2.57)	1.94 (1.69,2.20)	**< 0.001**	
LDL-C, mmol/L	2.99 (2.63,3.25)	2.86 (2.72,3.02)	**< 0.001**	
HDL-C, mmol/L	1.18 (1.00,1.42)	1.22 (1.05,1.44)	0.234	
ALT, IU/L	68.68 (39.56,102.04)	68.15 (40.64,96.10)	0.395	
AST, IU/L	77.67 (46.91,107.61)	81.99 (49.44,110.20)	0.416	
ALP, IU/L	113.00 (70.92,151.16)	113.47 (72.08,152.41)	0.691	
GGT, IU/L	43.41 (34.71,59.55)	43.93 (33.64,57.70)	0.781	
Serum CRP, mg/L	1.89 (1.40,2.34)	1.73 (1.23,2.22)	**0.002**	
Liver steatosis, n (%)	121 (53.5)	300 (37.7)	**< 0.001**	
CAP, dB/m	233 (211,264)	215 (189,258)	**< 0.001**	
Steatosis grade (%)				
S1	56.2	52.66	0.427	
S2	9.92	14.67		
S3	33.88	32.67		
Elastic modulus, kPa	11.8 (9.1,13.5)	11.0 (8.9,13.1)	0.125	
Liver fibrosis^***^, %	10.2/51.8/38.0	9.7/58.6/31.7	0.158	

Abbreviations here and below: HCV, hepatitis C virus; CKD, chronic kidney disease; BMI, body mass index; HOMA-IR, homeostasis model assessment-insulin resistance; eGFR, estimated glomerular filtration rate; TC, total cholesterol; TG, triglyceride; LDL-C, low-density lipoprotein cholesterol; HDL-C, high-density lipoprotein cholesterol; ALT, alanine aminotransferase; AST, aspartate aminotransferase; ALP, alkaline phosphatase; GGT, gamma-glutamyl transferase; CRP, C-reactive protein; CAP, controlled attenuation parameter

*Values are medians (IQRs) unless otherwise indicated

**Here and below, hypertension was defined as systolic blood pressure > 140 mmHg or diastolic blood pressure > 90 mmHg

***Liver fibrosis was classified as nil-mild/moderate-severe/cirrhosis based on E, as described in the Methods

***Genotype and allele frequencies of PNPLA3 rs738409 and TM6SF2 rs58542926*** in ***the CKD and non-CKD groups***.

The CKD and non-CKD groups had significant differences in the genotypes of *PNPLA3* rs738409 (*P* < 0.001) and in the genotypes of *TM6SF2* rs58542926 (*P* < 0.001) (Table 2). Analysis of allele frequencies indicated the CKD group had a higher frequency of the G allele in *PNPLA3* rs738409 (*P* < 0.001) and a lower frequency of the T allele in *TM6SF2* rs58542926 (*P* = 0.001).

**Table 2:** Genotype and allele frequencies of *PNPLA3* rs738409 and *TM6SF2* rs58542926 variants in the CKD and non-CKD groups

Variant	CKD, n (%)	non-CKD, n (%)	*χ* ^*2*^	*P* value
*PNPLA3* rs738409 Genotype				
CC	80 (35.4)	393 (49.4)	17.13	**< 0.001**
CG	117 (51.8)	347 (43.6)		
GG	29 (12.8)	56 (7.0)		
**Allele**				
C	277 (61.3)	1133 (71.2)	15.62	**< 0.001**
G	175 (38.7)	459 (28.8)		
***TM6SF2*** **rs58542926** **Genotype**				
CC	209 (92.5)	658 (82.7)	-	**< 0.001**
CT	15 (6.6)	136 (17.1)		
TT	2 (0.9)	2 (0.2)		
**Allele**				
C	433 (95.8)	1452 (91.2)	10.34	**0.001**
T	19 (4.2)	140 (8.8)		

### Relationship of PNPLA3 rs738409 and TM6SF2 rs58542926 variants with metabolic abnormalities in patients with CKD

**Fig. 1:**
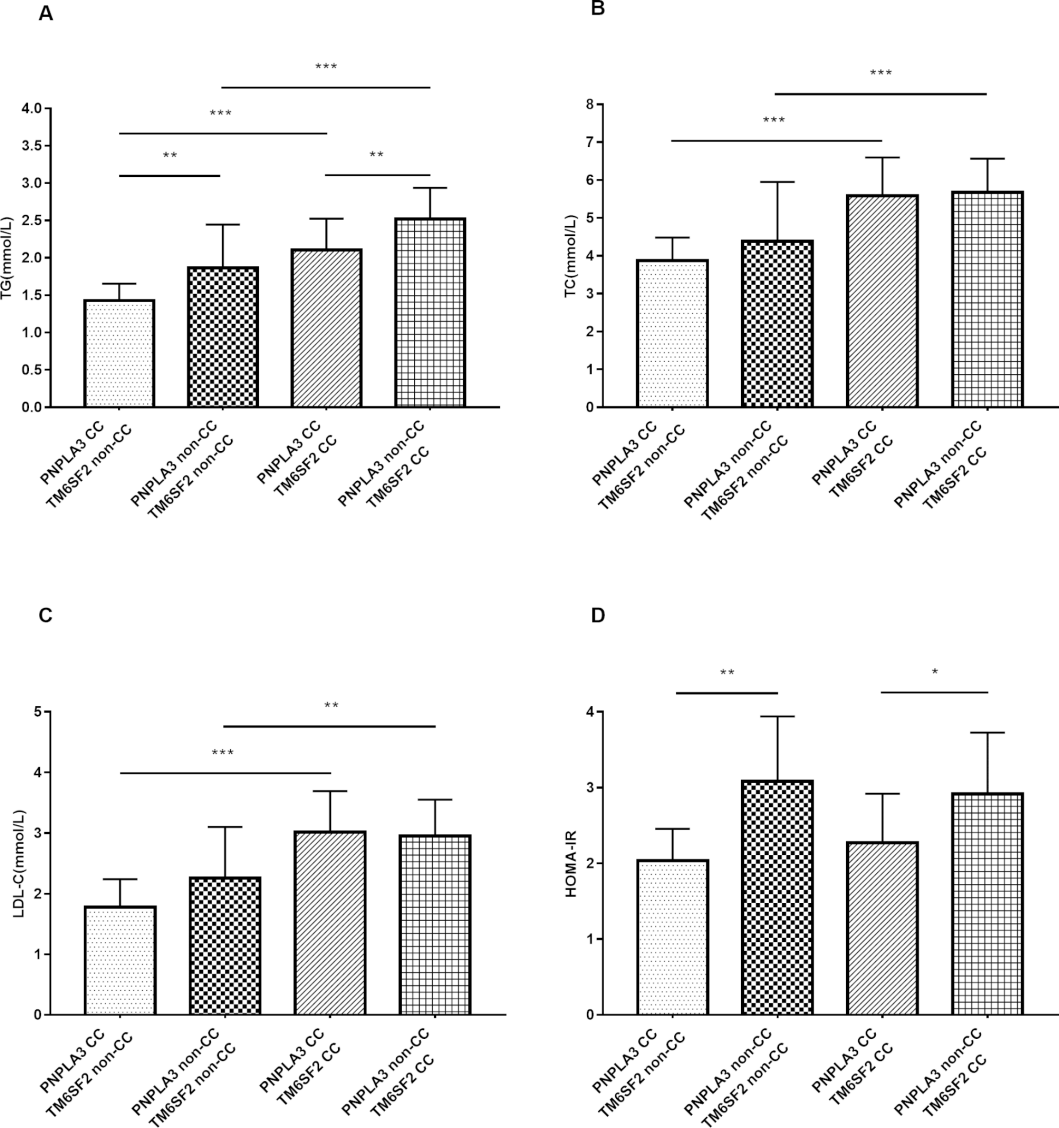
**Relationship of different combinations of** ***PNPLA3*** **rs738409 and** ***TM6SF2*** **rs58542926 variants with TGs (A), TC (B), LDL-C (C), and HOMA-IR (D) in patients with CKD (n = 226)** *P < 0.05, **P < 0.01, ***P < 0.001

The relationships of the combined presence of different variants of *PNPLA3* and *TM6SF2* with four different metabolic indicators in patients who had CKD were analyzed (Fig. 1). In general, patients who had *PNPLA3* rs738409 with the CC genotype had a better metabolic profile (lower TGs) than those who had *PNPLA3* rs738409 with the non-CC genotype. In addition, patients who had *TM6SF2* rs58542926 with the non-CC genotype had better metabolic profiles (lower TGs, TC, and LDL-C) than those had *TM6SF2* rs58542926 with the CC genotype. Notably, patients who had the *PNPLA3* rs738409 non-CC genotype had greater insulin resistance than those with the CC genotype, regardless of the *TM6SF2* genotype (Fig. 1D).

### Relationship of PNPLA3 rs738409 and TM6SF2 rs58542926 variants with eGFR in patients with CKD

**Fig. 2:**
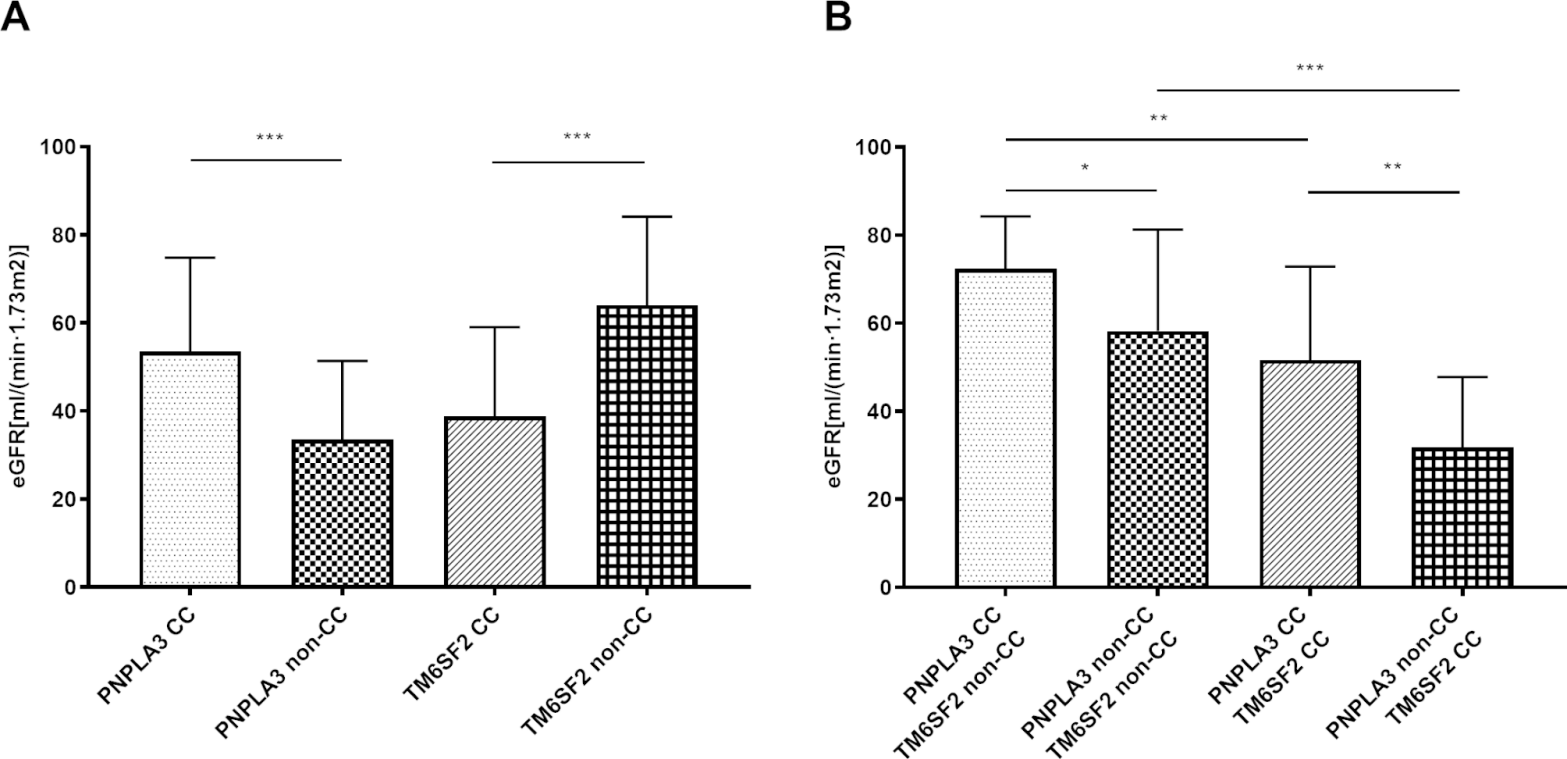
**Relationship of** ***PNPLA3*** **rs738409 and** ***TM6SF2*** **rs58542926 variants alone (A) and together (B) with eGFR in patients with CKD (n = 226)** *P < 0.05, **P < 0.01, ***P < 0.001

Next the relationships of eGFR with different individual variants and different combinations of variants were determined (Fig. 2). The results showed that the PNPLA3 G allele was associated with a lower eGFR, but that the TM6SF2 T allele was associated with a higher eGFR (both *P* < 0.001). Analysis of the effect of different combinations of variants showed that patients with the *PNPLA3* rs738409 CC genotype and the *TM6SF2* rs58542926 non-CC genotype had the highest eGFR, patients with the *PNPLA3* rs738409 non-CC genotype and the *TM6SF2* rs58542926 CC genotype had the lowest eGFR, and patients in the other two groups had intermediate eGFR.

### Relationship of PNPLA3 rs738409 and TM6SF2 rs58542926 variants with eGFR category in patients with CKD

**Fig. 3:**
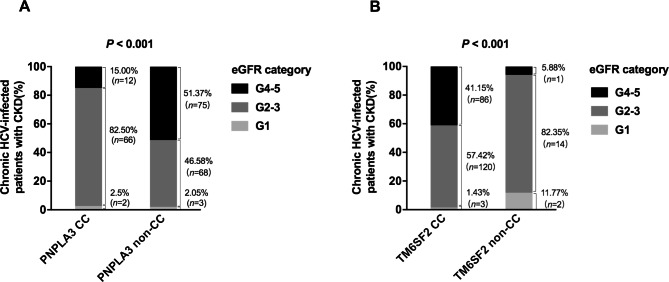
Relationship of *PNPLA3* rs738409 (A) and *TM6SF2* rs58542926 (B) variants with eGFR category in patients with CKD (n = 226)

The severity of CKD was associated with different *PNPLA3* and *TM6SF2* variants. Compared with patients who had *PNPLA3* CC, a greater proportion of non-CC patients had advanced CKD G4–5 (*P* < 0.001, Fig. 3A). Compared with patients who had *TM6SF2* non-CC, a greater proportion of CC patients had CKD G4–5 (*P* < 0.001, Fig. 3B).

### Factors related to the occurrence of CKD in patients with chronic HCV infections

Univariate and multivariate analyses of all 1022 patients were performed to identify factors associated CKD (Table 3). Univariate analysis revealed that high BMI (> 23 kg/m^2^), high HOMA-IR (≥ 2.5), hypertension, high CRP (> 2 mg/L), the *PNPLA3* rs738409 C > G variant, liver steatosis, high CAP, high TGs (≥ 1.7 mmol/L), high TC, and high LDL-C increased the risk for CKD, and the TM6SF2 rs58542926 C > T variant reduced the risk for CKD (all *P* < 0.05). Multivariate analysis confirmed that besides hypertension, high BMI, high HOMA-IR, and high serum TGs, liver steatosis and the *PNPLA3* rs738409 C > G variant remained associated with an increased risk of CKD, and the *TM6SF2* rs58542926 C > T variant remained associated with a decreased risk of CKD (all *P* < 0.05).

**Table 3:** Univariate and multivariate analyses of factors associated with CKD (n = 1022)

Variable	Univariate analysis		Multivariate analysis	
	OR (95%CI)	P	OR (95%CI)	*P* value
BMI > 23 kg/m^2^	2.23(1.61,3.09)	**< 0.001**	1.62(1.13,2.32)	**0.009**
HOMA-IR ≥ 2.5	2.25 (1.66,3.05)	**< 0.001**	1.71 (1.22,2.40)	**0.002**
Hypertension	4.29 (2.87,6.42)	**< 0.001**	4.06 (2.62,6.30)	**< 0.001**
TC	1.26 (1.07,1.48)	**0.006**	1.16 (0.97,1.39)	0.113
TG ≥ 1.7 mmol/L	2.66 (1.73,4.09)	**< 0.001**	2.30 (1.46,3.62)	**< 0.001**
CRP > 2 mg/L	1.42 (1.05,1.91)	**0.023**	1.34 (0.97,1.86)	0.078
LDL-C	1.41 (1.09,1.82)	**0.010**	1.26 (0.95,1.67)	0.116
HDL-C	0.78 (0.48,1.29)	0.341	0.68 (0.39,1.18)	0.170
CAP	1.01 (1.00,1.01)	**0.002**	1.00 (0.99,1.00)	0.224
Liver steatosis	1.91 (1.41,2.57)	**< 0.001**	2.11 (1.24,3.60)	**0.006**
*PNPLA3* C > G	1.78 (1.31,2.42)	**< 0.001**	1.65 (1.18,2.31)	**0.003**
*TM6SF2* C > T	0.39 (0.23,0.66)	**< 0.001**	0.40 (0.22,0.70)	**0.001**

Abbreviations: See Table 1

## Discussion

In this study, the analysis of 1022 patients with chronic non-genotype 3 HCV infections indicated that those who had CKD had higher prevalences of hypertension and liver steatosis, and higher levels of BMI, HOMA-IR, serum lipids, CAP, and CRP, but lower eGFR. Analysis of *PNPLA3* rs738409 variants indicated that patients with CKD had a lower proportion of the CC genotype, higher proportions of the CG and GG genotypes, and a higher frequency of the G allele. Analysis of *TM6SF2* rs58542926 variants indicated that patients with CKD had a higher prevalence of the CC genotype, lower prevalences of the CT and TT genotypes, and a lower frequency of the T allele. Moreover, patients in the CKD group with the *PNPLA3* rs738409 non-CC genotype had a higher TG level and increased insulin resistance, a higher prevalence of CKD G4–5, and lower eGFR, and those with the *TM6SF2* rs58542926 CC genotype had greater metabolic abnormalities, a higher prevalence of CKD G4–5, and a lower eGFR. Notably, liver steatosis and the *PNPLA3* rs738409 C > G variant were associated with an increased risk for CKD, whereas the *TM6SF2* rs58542926 C > T variant was associated with a reduced risk for CKD.

Chronic HCV infection leads to dyslipidemia and liver steatosis in most patients. For example, a retrospective study of 891 patients with chronic HCV infections reported that up to 70.5% of them had hyperlipidemia [31]. The incidence of steatosis in patients with chronic HCV infections ranges from 40 to 86%, higher than in individuals from the general population without HCV infections (20–30%), and also higher than in patients who have chronic HBV infections (~ 22%) or autoimmune liver disease (~ 16%) [32]. The incidence of steatosis in patients who have chronic HCV infections is related to viral factors and metabolic factors. Expression of the HCV genotype 3 core protein promotes steatosis and accumulation of large lipid droplets. Because the core protein of this genotype differs from that of other genotypes, and hepatic/blood viral load is related to steatosis in genotype 3 HCV-infected patients, this condition is called “viral steatosis”. However, for patients with non-genotype 3 HCV infections, steatosis is mainly related to metabolic factors (insulin resistance, lipid metabolic disorders, and oxidative stress) and it is called “metabolic steatosis” [33–35]. The HCV genotype 1 is predominant in China, so clinicians and researchers should focus on HCV-related metabolic abnormalities in Chinese patients. The examination of 1022 patients who had chronic HCV infections in this study indicated the prevalence of liver steatosis was 41.2%. None of these patients had HCV genotype 3, 645 (63%) had genotype 1, and 377 (37%) had genotype 2. This indicates that HCV infection complicated with liver steatosis in the study population should be mainly attributable to metabolic abnormalities.

In the present study demonstrated that the prevalences of dyslipidemia, liver steatosis, and high insulin resistance were significantly greater in patients with CKD than that in those without CKD. This suggests that abnormal metabolism contributes more to renal injury in these patients than previously believed. Further studies are needed to focus on whether HCV-related abnormal lipid metabolism is associated with the extrahepatic manifestations of HCV infection, especially renal injury.

The present analysis of the risk factors for CKD in patients with chronic non-genotype 3 HCV infections indicated that metabolic factors, such as increased BMI, insulin resistance, blood pressure, and TGs, increased the risk for renal injury. Notably, liver steatosis was an independent risk factor for renal injury.

The genetic risk factors for CKD and their association with dyslipidemia in patients with chronic non-genotype 3 HCV infections were also investigated. *PNPLA3* rs738409 and *TM6SF2* rs58542926 are two SNPs known to affect lipid metabolism. The results from previous studies of cultured cells, animal models, and patients confirmed that these two SNPs significantly affected lipid metabolism and accumulation [20–22, 24, 36–38]. Other research showed that the PNPLA3 protein has lipohydrolase activity, in that it promotes the hydrolysis of TGs [20], and that the TM6SF2 protein affects liver lipid metabolism by modulating the content of TG-rich lipoprotein and lipid droplets in the liver [22, 24, 36]. Valenti et al. [39] found that *PNPLA3* rs738409 I148M was associated with liver steatosis in patients with non-genotype 3 HCV infections, and was also independently associated with the severity of liver steatosis and the presence of liver fibrosis and cirrhosis. Two other studies [19, 40] showed that *TM6SF2* rs58542926 E167K increased the risk of liver steatosis in patients with non-genotype 3 HCV infections and was also associated with the severity of steatosis. Another study of NAFLD patients who did not have diabetes and were not obese showed that *PNPLA3* rs738409 I148M increased the risk for CKD, but *TM6SF2* rs58542926 E167K reduced the risk for CKD [25]. However, the relationships of these variants with CKD in patients who have chronic non-genotype 3 HCV infections have not yet been examined.

Comparison of patients with and without CKD indicated that the CKD group had a lower prevalence of the CC genotype and higher prevalences of the CG and GG genotypes of *PNPLA3* rs738409. The CKD group also had a higher prevalence of the CC genotype and lower prevalences of the CT and TT types of *TM6SF2* rs58542926. Multivariate analysis showed that the *PNPLA3* rs738409 C > G variant significantly and independently increased the risk for CKD, but the *TM6SF2* rs58542926 C > T variant significantly and independently reduced the risk for CKD. Previous studies showed that the *PNPLA3* rs738409 C > G variant reduced the hydrolysis of fatty acids and enhanced the transacylase activity of this enzyme for phospholipids, leading to the accumulation of fatty acids, TGs, and cholesterol in hepatocytes, and increased insulin resistance [20, 41, 42]. Other research showed that the *TM6SF2* rs58542926 C > T variant led to decreased secretion of very low density lipoprotein in the liver, leading to reduced secretion of TGs and TC in hepatocytes, the accumulation of lipid droplets in hepatocytes, and related diseases [22, 24, 36]. The present study of patients with non-genotype 3 HCV infections showed that patients with the *PNPLA3* rs738409 non-CC genotype had higher serum levels of TGs and insulin resistance than those with the CC genotype, and that the serum TGs, TC, and LDL-C levels were lower in patients with the *TM6SF2* rs58542926 non-CC genotype than in those with the CC genotype. This supports the hypothesis that different variants of *PNPLA3* and *TM6SF2* affect the level of circulating blood lipids and insulin resistance, and that these metabolic alterations affect the risk for CKD.

This study further confirmed that compared to those with *PNPLA3* rs738409 CC, individuals with *PNPLA3* rs738409 C > G had an increased risk of CKD, a lower eGFR, and were more likely to have advanced CKD G4–5; compared to those with *TM6SF2* rs58542926 CC, individuals with *TM6SF2* rs58542926 C > T had a decreased risk of CKD, a higher eGFR, and were less likely to have CKD G4–5. These findings suggest that detection of genetic risk factors may help predict the occurrence and progression of CKD. A clinical cohort study of the relationships of DAA treatment with *PNPLA3* rs738409 and *TM6SF2* rs58542926-induced dyslipidemia as well as the improvement of renal function in patients with non-genotype 3 chronic HCV infection is currently in progress (to be published later).

A lipid metabolism disorder may induce renal injury by several possible mechanisms. First, it can induce insulin resistance by inhibiting pathways related to renal insulin signaling [43]. Second, it can cause mesangial cell proliferation, extracellular matrix accumulation, endothelial cell dysfunction, and epithelial cell damage, and thus directly or indirectly promote the progression of injury to the renal tubulointerstitium due to peroxidation-mediated damage of renal tubular epithelial cells [44–46]. Third, it can promote the occurrence and development of glomerulosclerosis by damaging renal microvessels [47, 48]. A lipid metabolism disorder cannot simply lead to abnormal lipid deposition in the kidneys, but can also lead to liver steatosis, a condition that aggravates blood lipid disorders and renal injury, induces insulin resistance, and promotes the release of pro-inflammatory factors and oxidative stress [49].

### Study strengths and limitations

This study analyzed the relationships of *PNPLA3* rs738409 and *TM6SF2* rs58542926 gene polymorphisms with CKD in patients with chronic HCV infections. This study is the first to find that metabolic disorders induced by genetic factors in patients are important causes of CKD in those with non-genotype 3 HCV infections. However, this study was only conducted in a Han population from Northeast China, and the conclusions may not be applicable to patients from other regions and different ethnicities. Moreover, the occurrence of CKD may lead to lipid metabolism disorders. Therefore, further clinical studies with broader coverage and larger sample sizes are needed to confirm the conclusions presented here and to provide in-depth analysis of the underlying mechanism.

## Conclusions

In conclusion, the major results of this study are that lipid metabolism disorders and liver steatosis contribute to CKD in patients who have chronic non-genotype 3 HCV infections. This study also found that two SNPs — *PNPLA3* rs738409 and *TM6SF2* rs58542926 — that have known effects on lipid metabolism and deposition in the liver, are genetic risk factors for CKD in these patients. Importantly, these two SNPs have different affects on the development of CKD. Further study of the *PNPLA3* rs738409 and *TM6SF2* rs58542926 variants may help elucidate the mechanism of CKD in patients who have chronic non-genotype 3 HCV infections. Investigating the *PNPLA3* rs738409 and *TM6SF2* rs58542926 variants helps to elucidate the etiology of liver steatosis in HCV-infected patients, and also helps to assess the relationship between HCV infection and CKD. The results presented here therefore may be helpful to high-risk individuals by enabling them to take prompt and proactive measures to correct metabolic disorders and for anti-HCV treatment, thus improving the outcomes.

## Data Availability

The datasets generated and analysed during the current study are not publicly available due to limitations of ethical approval involving the patient data and anonymity but are available from the corresponding author on reasonable request.

## Abbreviations

HCV: Hepatitis C virus
CKD: Chronic kidney disease
DAA: Direct-acting antiviral
NAFLD: Non-alcoholic fatty liver disease
SNP: Single nucleotide polymorphism
PNPLA3: Patatin-like phospholipase domain-containing protein 3
TM6SF2: Transmembrane 6 superfamily member 2
HBV: Hepatitis B virus
AER: Albumin excretion rate
CKD-EPI: Chronic Kidney Disease Epidemiology Collaborative
CT: Computed tomography
HWE: Hardy-Weinberg equilibrium
BMI: Body mass index
FINS: Fasting insulin
HOMA-IR: Homeostasis model assessment-insulin resistance
eGFR: Estimated glomerular filtration rate
TC: total cholesterol
TG: Triglyceride
LDL-C: Low-density lipoprotein cholesterol
HDL-C: High-density lipoprotein cholesterol
ALT: Alanine aminotransferase
AST: Aspartate aminotransferase
ALP: Alkaline phosphatase
GGT: Gamma-glutamyl transferase
CRP: C-reactive protein
CAP: Controlled attenuation parameter
